# Downstream Targets of VHL/HIF-α Signaling in Renal Clear Cell Carcinoma Progression: Mechanisms and Therapeutic Relevance

**DOI:** 10.3390/cancers15041316

**Published:** 2023-02-19

**Authors:** Sonia Mazumder, Paul J. Higgins, Rohan Samarakoon

**Affiliations:** Department of Regenerative & Cancer Cell Biology, Albany Medical College, Albany, NY 12208, USA

**Keywords:** ccRCC, HIF-α, VHL, EGFR, MET

## Abstract

**Simple Summary:**

Clear cell renal cell carcinoma (ccRCC) progression, which is the most common form of kidney cancer, is associated with the loss of the Von Hippel-Lindau (VHL) gene in renal epithelium. VHL promotes proteasomal degradation of Hypoxia Inducible Factor alpha (HIF-α) thereby inhibiting the transcription of genes that are required for cell growth and proliferation during hypoxia (lack of oxygen). Accumulation of HIF-α consequent to VHL loss results in the activation of HIF target genes irrespective of oxygen availability which initiates tumorigenesis in the kidney. In this review, we discuss which signaling networks and genes are upregulated in response to HIF-α activation and how they drive ccRCC progression. We also highlight the potential therapies available to treat ccRCC patients as well as several medical and scientific challenges that impede the scientific investigation in this field.

**Abstract:**

The clear cell variant of renal cell carcinoma (ccRCC) is the most common renal epithelial malignancy and responsible for most of the deaths from kidney cancer. Patients carrying inactivating mutations in the Von Hippel-Lindau (VHL) gene have an increased proclivity to develop several types of tumors including ccRCC. Normally, the Hypoxia Inducible Factor alpha (HIF-α) subunits of the HIF heterodimeric transcription factor complex are regulated by oxygen-dependent prolyl-hydroxylation, VHL-mediated ubiquitination and proteasomal degradation. Loss of pVHL function results in elevated levels of HIF-α due to increased stability, leading to RCC progression. While HIF-1α acts as a tumor suppressor, HIF-2α promotes oncogenic potential by driving tumor progression and metastasis through activation of hypoxia-sensitive signaling pathways and overexpression of HIF-2α target genes. One strategy to suppress ccRCC aggressiveness is directed at inhibition of HIF-2α and the associated molecular pathways leading to cell proliferation, angiogenesis, and metastasis. Indeed, clinical and pre-clinical data demonstrated the effectiveness of HIF-2α targeted therapy in attenuating ccRCC progression. This review focuses on the signaling pathways and the involved genes (cyclin D, c-Myc, VEGF-a, EGFR, TGF-α, GLUT-1) that confer oncogenic potential downstream of the VHL-HIF-2α signaling axis in ccRCC. Discussed as well are current treatment options (including receptor tyrosine kinase inhibitors such as sunitinib), the medical challenges (high prevalence of metastasis at the time of diagnosis, refractory nature of advanced disease to current treatment options), scientific challenges and future directions.

## 1. Introduction

### 1.1. Renal Clear Cell Carcinoma (ccRCC)

Renal cell carcinoma (RCC) is a common urologic malignancy, which accounts for the majority (80%) of kidney cancers worldwide [1,2]. Several subtypes are evident based on histopathologic and molecular criteria including clear cell renal cell carcinoma (ccRCC), which is the most prevalent (constituting 70% of all renal tumors), and responsible for majority of the deaths from kidney cancer. Approximately 10–15% of renal malignancies are classified as papillary RCCs; chromophobe RCCs comprise 5%; and less than 1% are collecting duct RCCs [1,3]. Clear cell carcinomas originate mainly in the proximal tubule and have a typical yellow surface due to a high lipid content [3]. The name ccRCC is derived from the distinct clear, and translucent morphology of the cytoplasmic compartment. This appearance is due to the abundance of glycogen and lipids which occupy most of the cytoplasmic space [1,3,4,5,6].

### 1.2. RCC Statistics

Among the malignant neoplasms, RCC is the 12th most prevalent worldwide and ranks 9th in the United States [7]. RCC is increasing throughout the world in recent years and the incidence in developed countries is particularly higher [7]. The highest rates of kidney cancer are in Europe and North America [7,8]. A dramatic increase in the five-year survival rate is evident over the last 60 years [9], due in part to the advances in the early detection RCC lesions and the availability of improved surgical and ablative techniques as well as advanced systemic treatment options for patients with locally advanced or metastatic RCC [10]. According to GLOBOCAN data, an estimated 431,288 new cases of renal cancer were diagnosed in 2020 in approximately 271,000 men and 160,000 women and disease related deaths involved 115,600 men and 63,768 women. The occurrence of RCC is more common in males (1.5 times) than females. The average age standardized rate (ASR) in 2020 was 4.6 globally with a rate of 6.1 for men and 3.1 for women. North America had the highest ASR at 12.2, while ASRs in Asia and Africa were lower at 2.8 and 1.8, respectively [11].

Kidney cancer incidence differs based on ethnicity as well. In the United States, African Americans are more prone to develop renal malignancies than the white population despite having similar mortality rates [8]. Asian Americans or Pacific Islanders have the lowest tendency to develop renal cancers compared to other ethnic groups in the US [9]. In 2022, approximately 79,000 new cases of kidney cancer were diagnosed in the US, accounting for 4.1% of all cancers. Estimated RCC deaths were 13,920 (comprising 2.3% of all cancer deaths in 2022) with a 5-year survival rate of 76.5% [12,13]. According to the American Cancer Society’s most recent data (2023), among the 81,800 new cases with kidney cancer, 52,360 are in men and 29,440 in women in the US; about 14,890 people (9920 men and 4970 women) will die from this disease [14].

### 1.3. Risk Factors Associated with ccRCC

Genetic factors are major contributors to the development of RCC. ccRCC is often a clinical manifestation of Von Hippel-Lindau (VHL) disease, a rare, autosomal dominant and familial cancer syndrome caused by the inactivation of a single VHL allele (by mutation, deletion, or hypermethylation) resulting in loss of VHL function [15,16]. The incidence of RCC increases with age [17,18] with men more likely to be affected than women [17,19]. Established risk factors associated with the development of ccRCC include tobacco use, hypertension, obesity and acquired kidney diseases [2,17,19,20,21,22,23]. Cigarette smoking, in fact, doubles the risk of RCC compared to non-smokers [21,24,25]. Several conditions are also associated with renal cancer including a family history of chronic and cystic kidney diseases, renal transplantation, kidney tuberous sclerosis and possibly diabetes mellitus [17,21,26,27,28,29].

## 2. Mechanism of Loss of Function of VHL Gene in ccRCC

Von Hippel–Lindau (VHL) is a tumor suppressor gene located on chromosome 3p25 [30]. Mutation in one allele results in the formation of VHL disease [19]. An inactivating mutation in the remaining VHL allele is a driving force for the development of ccRCC, a common associated secondary lesion of the Von Hippel–Lindau (VHL) deficiency syndrome [1,31]. Biallelic VHL gene inactivation in ccRCC can occur via point mutation, indels (insertion or deletion mutations), loss of alleles on chromosome 3p25 and epigenetic alterations including hypermethylation of the VHL gene promoter resulting in a loss of the tumor suppressor function of pVHL [4,31,32,33,34,35,36].

### 2.1. Consequences of VHL Loss—Stabilization and Accumulation of HIF-α

**Figure 1:**
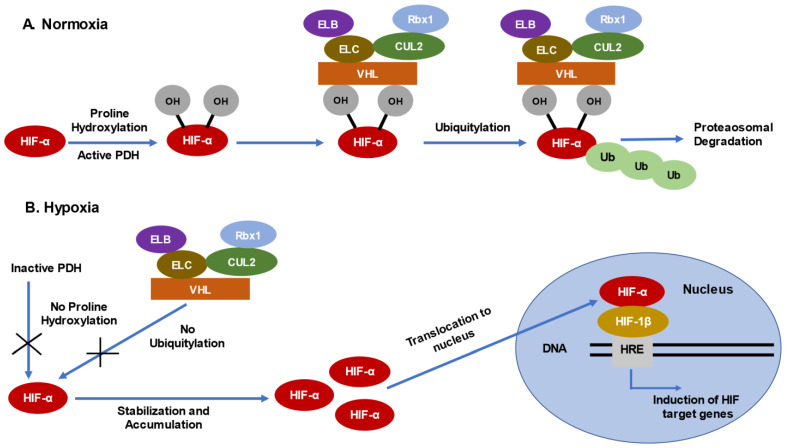
Regulation of HIF-α via VHL-dependent ubiquitylation and proteasomal degradation. (**A**) Alpha subunits of the Hypoxia Inducible Factors (HIFs) are constitutively expressed but are tightly regulated. Under normal oxygen availability, HIF-α subunits are hydroxylated at specific proline residues via the activity of Proline Hydroxylases (PDHs), which target HIF-α for degradation via VHL- dependent recognition and ubiquitination. (**B**) Under low oxygen availability, however, PDH-dependent hydroxylation of proline residues in HIF-α is absent; as a result, recognition via VHL and subsequent HIF-α degradation is inhibited. Consequently, HIF-α subunits are stabilized, accumulate, and translocate to the nucleus, forming heterodimeric complexes with HIF-1β subunits. This complex interacts with the (HIF Response Element) HRE in DNA to induce the expression of HIF target genes that regulate angiogenesis, cell growth, cell cycle progression, cell proliferation, glycolysis, and apoptosis to maintain cell growth and survival under hypoxic conditions.

pVHL is a component of the mammalian Skp1-cullin 1-F-box (SCF-2) E3 ubiquitin ligase complex consisting of Cullin-2 (Cul-2), Elongins B/C (ELB/ELC), and the RING finger protein (Rbx-1) and is a receptor subunit of the E3 Ubiquitin ligase complex that targets several substrates for ubiquitination and proteasomal degradation [37,38,39,40,41,42]. pVHL plays a critical role in cellular oxygen sensing where it regulates the activity of HIFs under normoxic conditions. In a normoxia environment, HIF-1α and HIF-2α are constantly synthesized and degraded, due to the activity of proline hydroxylases (PHD-1, PHD-2, PHD-3) which, in the presence of oxygen, hydroxylate HIF-α subunits at specific proline residues. This hydroxylation facilitates recognition by pVHL [43]. pVHL, in turn, ubiquitinates the HIF-α subunits, facilitating their degradation in the presence of oxygen [44,45,46] (Figure 1). This mechanism ensures that HIF transcription factors accumulate only under low oxygen (hypoxic) conditions.

Loss of pVHL leads to HIF-α stabilization and accumulation even in a normoxic environment, resulting in activation of HIF target genes (Figure 1) that, collectively, impact several cellular, physiologic and pathophysiologic processes such as angiogenesis, cell growth, cell cycle progression and proliferation, glycolysis and apoptosis [47]. Indeed, ccRCC is a highly angiogenic malignancy due to the fact that the tumor cells overproduce hypoxia inducible VEGFA mRNAs in the absence of VHL, irrespective of oxygen availability [48,49]. This highly angiogenic behavior can be attenuated by reintroducing wild-type pVHL, while the expression of mutated pVHL fails to rescue this function in ccRCC [50,51,52]. These studies suggest that loss of pVHL results in the induction of HIF-responsive genes in ccRCC progression.

### 2.2. HIF-α Involvement in ccRCC—Contrasting Role of HIF-1α and HIF-2α in ccRCC

**Figure 2:**
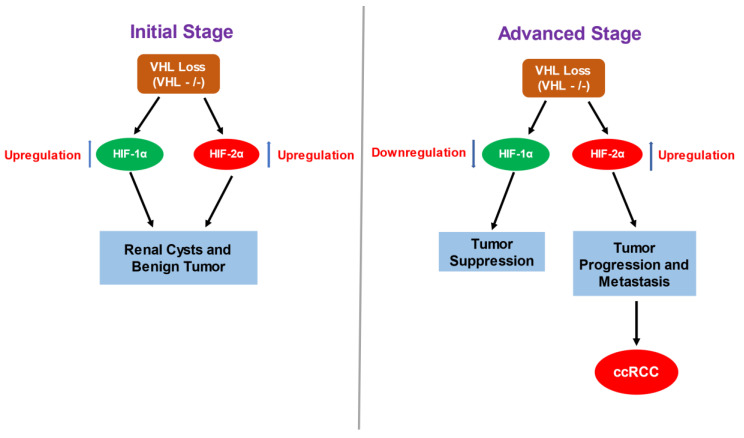
Contrasting Role of HIF-1α and HIF-2α in ccRCC progression. Loss of VHL leads to the upregulation of HIF-α isoforms: HIF-1α and HIF-2α, irrespective of oxygen availability in cell, which in turn contributes to the formation of initial renal cysts and tumors. Interestingly, only the HIF-1α isoform is expressed in normal tubular cells and the initiation of HIF-2α expression upon VHL loss, acts as an initial trigger for transformation of healthy tubular cells. Overtime, the expression of HIF-2α prevails over HIF-1α expression, which is associated with tumor growth, progression and metastasis. Furthermore, HIF-1α attenuates expression of several pro-tumorigenic genes which otherwise are stimulated by HIF-2α suppressing the malignant phenotype.

Almost all the RCC-associated mutations in VHL are defective in recognizing and degrading HIF-α subunits leading to accumulation of HIF-α and overexpression of HIF target genes [53]. Although initial VHL inactivation leads to the expression and accumulation of both HIF-1α and HIF-2α, with time, HIF-2α is predominant and suppresses HIF-1α at the protein level [54]. Indeed, the formation of both renal cysts and tumors was prevented upon inactivation of HIF-1α and HIF-2α in VHL-deficient mice, indicating their shared involvement during the initial stages of ccRCC development [55] whereas only the HIF-1α isoform was evident in normal tubular cells [56,57]. These findings suggest that the initiation of HIF-2α expression occurs upon pVHL loss-of-function and acts as a trigger in the transformation of tubular epithelial cells (Figure 2). A polymorphism linked to HIF-2α, in fact, was identified as a ccRCC risk factor in a genome-wide association study [58]. The activity of HIF-α isoforms is cell-context specific where HIF-1α accumulates and functions during the initial (2–24 h) response to acute hypoxia which decreases rapidly with prolonged exposure [59]. HIF-2α is predominant under chronic (>24 h) hypoxic conditions, resulting in a switch from HIF-1α to HIF-2α activity [59]. A similar phenomenon takes place during the progression of ccRCC, where HIF-2α eventually predominates over HIF-1α to promote tumor growth and metastasis [54].

Besides the key oxygen dependent pVHL mediated regulation of HIF-α subunits, several oxygen- and pVHL-independent mechanisms contribute to the HIF switch. Receptor of activated protein kinase C (RACK1) competes with heat shock protein 90 (HSP90) for binding to HIF-1α (which otherwise stabilizes it), thereby promoting its ubiquitination and degradation by recruiting Elongin C [60]. Kruppel-like Factor 2 (KLF2) also disrupts the interaction between HIF-1α and HSP 90 selectively inducing pVHL-independent but proteasome-dependent degradation of HIF-1α in the endothelium; KLF2, in contrast, has no effect on HIF-2α stability [61]. The HSP70/CHIP complex is an additional factor that specifically ubiquitinates HIF-1α, but not HIF-2α, causing its proteasomal degradation [62]. Human double minute 2 (hdm2) protein also mediates HIF-1α degradation in a p53 dependent manner [63]. Importantly, hypoxia-associated factor (HAF, also known as SART1800 or squamous cell carcinoma antigen recognized by T cells) is an E3 ubiquitin ligase that mediates the HIF-1α to HIF-2α switch during ccRCC progression. Mechanistically, HAF binds to and preferentially ubiquitinates HIF-1α by an oxygen- and pVHL-independent mechanism to target it for proteasomal degradation [64,65] while promoting the transactivation of HIF-2α via hypoxia-induced HAF SUMOylation [65,66]. Ectopic expression of HAF, indeed, reduces the activity of HIF-1α and increases ccRCC growth and metastasis in vivo [64]. micro RNAs (miRNAs) as well as long non-coding RNAs (lncRNAs) are also involved in regulating the HIF-1α to HIF-2α transition [67]. miR-18a activity, for example, is markedly induced during prolonged hypoxia and directly target HIF-1α mRNA, contributing to a HIF switch [68]. A recent study in human endothelial cells identified the oxygen redistribution-dependent reactivation of PHD-3 (Prolyl Hydroxylase-3) [69] which has a higher specificity for HIF-1α isoform for hydroxylation and pVHL dependent ubiquitination and degradation [70]. This oxygen redistribution-dependent PHD-3 reactivation leads to HIF-1α mRNA destabilization as a mechanism of HIF switch during the extended hypoxic response [69]. Collectively, these mechanisms favor accumulation of HIF-2α, which mediates RCC progression.

Increased tumor growth is also associated with overexpression of HIF-2α in a mouse ccRCC-xenograft model [53,54] and RNA interference-mediated knockdown of HIF-2α reduced tumor growth [71]. Inhibition of HIF-2α, moreover, is sufficient for growth suppression in VHL^−/−^ malignancies [72]. In sharp contrast, elevated expression of HIF-1α reduced tumor size whereas HIF-1α knockdown increased cell proliferation [54,73]. Additionally, induction of HIF-2α in a pVHL-reconstituted pre-clinical ccRCC model attenuated xenograft growth while re-expression of HIF-1α was without effect [16,53,54].

These observations collectively suggest a tumor suppressive role for HIF-1α and the oncogenic potential of HIF-2α. Indeed, HIF-1α exerts negative effects on pro-tumorigenic genes including cyclin D1, transforming growth factor alpha (TGF-α), and vascular endothelial growth factor (VEGF) while these genes are stimulated by HIF-2α to promote a malignant phenotype [54]. Expression of HIF-2α target genes activate diverse signaling pathways leading to ccRCC tumorigenesis, which will be the focus of this review.

## 3. HIF-2α Target Genes and HIF-2α-Driven Signaling Pathways Implicated in ccRCC Progression and Metastasis

### 3.1. TGF-α and EGFR

**Figure 3:**
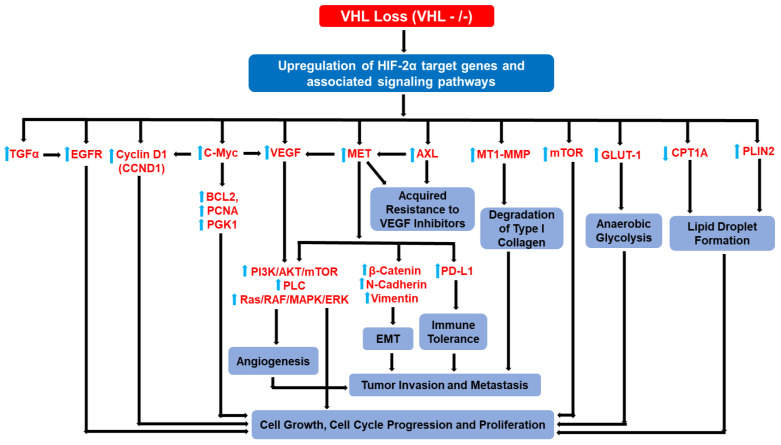
HIF-2α-induced genetic responses and the associated oncogenic hierarchical events leading to ccRCC progression. HIF-2α induces the expression of multiple target genes (e.g., EGFR, CCND1, c-Myc, MET, VEGF, mTOR, GLUT1, CPTA1). Such gene reprogramming events promote angiogenesis, cell proliferation/growth, cell cycle progression, EMT, tumor migration/invasion, anaerobic glycolysis, lipid accumulation, immune tolerance, and drug resistance, which collectively lead to advanced ccRCC. Various functional interplay among target proteins and downstream pathogenic consequences are depicted above. VEGF-mediated tumor angiogenesis, for example, can promote tumor invasion and growth. Similarly, metabolic reprogramming (e.g., glycolysis induction) initiated by Glut1 can sustain cancer growth.

Increased epidermal growth factor receptor (EGFR) activity is common in human ccRCC and is related to a poor prognosis, supporting the possible involvement of the EGFR in ccRCC progression [74,75,76,77]. Elevated HIF consequent to VHL loss upregulates TGF-α, an EGFR ligand, in ccRCC tumors and cell lines while reintroduction of wild type pVHL attenuates TGF-α expression in ccRCC cells [78,79]. Inhibition of TGF-α production in VHL^−/−^ RCC cells, moreover, reduces cell growth in culture [80] suggesting that TGF-α is important for ccRCC growth and proliferation. TGF-α exerts its effect on cell proliferation by stimulating EGFR dependent cell signaling leading to ccRCC tumor growth [78,80,81] (Figure 3). EGFR silencing efficiently blocked HIF-2α mediated tumorigenesis in VHL^−/−^ RCC cell lines indicating that HIF-2α is a major factor in the TGF-α/EGFR dependent pathway of renal carcinogenesis [82].

HIF-2α can also upregulate EGFR expression by stimulating formation of a complex with the RNA-binding protein RBM4 and the cap-binding eIF4E2 which then occupy the HIF-2α binding site on EGFR transcripts targeting EGFR mRNA to the polyribosome [83,84]. HIF-2α prolongs EGFR activity, moreover, by inhibiting its endocytosis and subsequent lysosomal degradation in VHL-deficient 786-O ccRCC cell lines [76]. The activated (phosphorylated) EGFR is more stable in VHL-deficient ccRCC cells compared to pVHL-positive ccRCC cells, since pEGFR is degraded via VHL-mediated proteasomal mechanisms which are significantly attenuated upon VHL loss in ccRCC tumors [77].

### 3.2. c-Myc

c-Myc, a proto-oncogene, promotes cell cycle progression from G1 to S phase by regulating the activity of several cyclins and CKIs (CDK inhibitors) [85] which are elevated in many cancers [86,87]. The increased expression of c-Myc as well as its target genes in VHL-deficient ccRCCs, implies a pivotal role in tumor progression [88]. Indeed, the upregulation of 37 different MYC-regulated genes including BCL2, CCND1, PCNA, PGK1, and VEGFA, all promote tumor formation [87,89,90,91,92,93,94,95,96]. HIF-2α elevates c-Myc activity, inducing cell cycle progression in ccRCC cells (Figure 3). In contrast, HIF-1α inhibits c-Myc growth responsive genes, decreasing the level of E2F and cyclin D2, while enhancing the expression of the cell cycle progression inhibitors p21 and p27 [97,98]. Knockdown of Myc expression attenuates growth and induces G0/G1 phase cell cycle arrest in ccRCC cells, correlating with the reduced expression of Myc-target genes [88]. The HIF-2α-c-Myc pathway, furthermore, reduces replication stress by promoting homologous recombination (HR)-mediated DNA repair as well as reducing checkpoint responses to ensure uninterrupted cell proliferation [88,97]. These findings suggest that upregulation of c-Myc activity is critical for ccRCC progression.

### 3.3. VEGF

Hypoxia is a master regulator of angiogenesis and aberrant angiogenesis is a key aspect of several pathological conditions including solid tumors. The HIF network regulates the expression of multiple pro-angiogenic genes including vascular endothelial growth factor (VEGF), angiopoietin-1, angiopoietin-2, platelet-derived growth factor (PDGF), basic fibroblast growth factor (bFGF) [99]. Indeed, ectopic stimulation of the HIF pathway is sufficient to induce localized angiogenesis, even in the absence of these pro-angiogenic factors [100]. Tumor induced hypoxia contributes to enhanced expression of these factors in order to induce the HIF-specific angiogenic program. Mechanistically, HIF signaling network increases vascular permeability, stimulates the recruitment of endothelial progenitor cells (EPCs) and their differentiation to endothelial cells (ECs), induces the expression of enzymes (e.g., MMPs) required for the sprouting and splitting of the pre-existing blood vessels as well as induces localization of the supporting cells such as smooth muscle cells and pericytes for blood vessel maturation as a final step [101].

VEGF acts as a potent angiogenic inducer during tumor progression and metastasis and binds to tyrosine kinase receptors, including VEGFR1 or VEGFR2, on the vascular endothelial cell surface [102]. VEGFA is highly expressed in ccRCC tumors upon pVHL loss-of-function (Figure 3) indicating its hyper-vascular nature. VEGF overexpression corelates with ccRCC clinical stage, tumor grade, lymph node metastasis as well as overall survival [49,103,104,105,106]. Indeed, pharmacological suppression of VEGF in different VHL^−/−^ ccRCC cell lines impairs xenograft growth in nude mice, implicating VEGF as a potent tumor-promoting gene in ccRCC. HIF-2α is an upstream regulator of VEGF expression in ccRCC and RNA interference of HIF-2α in 786-O VHL^−/−^ cells mitigated VEGF expression suggesting that VEGF is a potential HIF-2α target in ccRCC [107,108,109]. Several anti-angiogenic therapies are available to disrupt the VEGF signaling axis in metastatic ccRCC including bevacizumab [110,111], a monoclonal antibody that targets VEGFA and sunitinib [112], sorafenib [113], and tyrosine-kinase inhibitors (TKIs) target VEGFR2. However, chronic treatment contributes to TKI resistance due to underlying crosstalk between VEGFR and other downstream targets of the receptor [114].

### 3.4. Cyclin D1

The G1/S transition, mediated by Cyclin D1 (encoded by the CCND1 gene), is evident in many malignancies stressing its importance in the transformation process [115,116,117,118,119,120,121,122,123]. A VHL regulated gene, Cyclin D1 is upregulated upon VHL inactivation while reintroduction of pVHL reduced Cyclin D1 levels [124,125]. CCND1 is significantly elevated both at the transcriptional and translational levels in ccRCC tumors [54,107,126,127,128]. Increased Cyclin D1 levels in RCC correlated with disease progression [129]. The causative role of Cyclin D1 in ccRCC tumor progression is highlighted by the fact that CCND1 knockdown in VHL deficient ccRCC cell lines attenuates xenograft growth in vivo [107]. Moreover, HIF-2α is sufficient to maintain tumor growth in VHL-deficient kidney cancer cells [53,71] via Cyclin D1 activation [54].

Indeed, CCND1 is a specific HIF-2α response gene since CCND1 decreases upon HIF-2α inhibition and increases following re-expression of HIF-2α in vitro [54] (Figure 3). Ectopic expression of HIF-1α in 786-O cells, conversely, suppresses CCND1 levels [54]. Additionally, the HIF-2α antagonist, PT2399, reduces CCND1 levels in pre-clinical kidney cancer models [108]. Importantly, genetic polymorphism at the 11q13.3 locus, which causes predisposition to RCC development, affects HIF-2α dependent induction of CCND1 expression by allowing HIF-2α to bind to the enhancer region of Cyclin D1, a phenomenon specifically restricted to VHL-deficient RCC [130]. The pro-oncogenic CDK4/6-cyclin D/Rb signaling axis can be targeted by Ribociclib (an oral cyclin-dependent kinase 4 and 6 (CDK4/6) inhibitor), which potently attenuates cell proliferation in RCC pre-clinical models [131]. An in silico study recently identified rutin and curcumin, which specifically bind to CCND1 and inhibit its association with CDK4/CDK6 complex, which is required for G1-S phase transition [132], highlighting the potential to disrupt Cyclin D1 signaling pathway during RCC progression.

### 3.5. GLUT1

Metabolic reprogramming in cancer cells accommodates the increased requirements for glucose, amino acids, lipids, and nucleotides to support proliferation [133,134]. The glycolytic pathway is dysregulated in ccRCC patients with a metabolic switch from aerobic glycolysis and mitochondrial oxidative phosphorylation to an anaerobic pathway [135,136,137]. The expression of glucose transporter-1 (GLUT-1) and enolase 2 (ENO2) is induced by HIF-2α in ccRCC [53,54,107], suggesting a causative role in this metabolic switch (Figure 3). Glucose deprivation or blocking the activity of GLUT-1 attenuates tumor formation and apoptosis of VHL-deficient RCC cells [138]. High GLUT-1 mRNA levels positively correlate with the tumor grade and poor prognosis in ccRCC patients [139]. HIF-2α induced expression of GLUT-1 is Nox4 dependent since genetic inhibition of Nox4 not only represses HIF-2α transcription but also abrogates GLUT-1 gene induction in 786-O cells, possibly linking NOX4-induced oxidative stress response to the HIF-2α-GLUT1 signaling axis in RCC progression [140]. Moreover, elevated level of GLUT-1 transporter is linked to poor CD8^+^ effector T cells infiltration in the tumor, suggesting that targeting upregulation of GLUT-1 could improve immunotherapeutic efficacy and induce cancer cell death [141].

Several miRNAs have recently been identified as upstream mediators of GLUT1 expression during RCC. miR-1291 is dramatically downregulated in RCC, and ectopic expression of miR-1291 in A498 and 786-O cells reduces proliferation by suppressing the SLC2A1/GLUT1 axis [142]. Moreover, in several malignancies, GLUT-1 mRNA can be repressed by hsa-miRNA-144 as well as hsa-miRNA-186 [143,144] and an inverse association between these miRNAs and GLUT-1 mRNA levels is evident during ccRCC [145]. While mechanisms are not fully understood, targeting certain miRNAs could be an indirect strategy to reduce GLUT1 expression and attenuate glycolytic reprogramming events linked to RCC growth.

### 3.6. Carnitine Palmitoyltransferase 1A (CPT1A) and Perilipin 2 (PLIN2)

High lipid accumulation in ccRCC imparts its clear cell morphology [3] suggesting the reduced catabolism of lipids. The formation of lipid droplets is negatively regulated via a VHL-dependent mechanism and, conversely, VHL loss induces the formation of lipid droplets in ccRCC cell lines (RCC4, RCC10, and 786-O). Indeed, reintroduction of VHL into RCC4, RCC10, and 786-O ccRCC cells dramatically decreased lipid deposition in vitro and in vivo. Moreover, genetic knockdown of both HIF-1α and HIF-2α attenuates lipid droplet formation in RCC4 cells suggesting an involvement of HIF-α in lipid deposition in ccRCC. Carnitine palmitoyltransferase 1A (CPT1A) is a rate-limiting enzyme which controls fatty acid (FA) transport into the mitochondria and promotes FA beta-oxidation. Indeed, CPT1A is a direct target of HIF-α and its expression is repressed by both HIF-1α and HIF-2α (Figure 3). HIF-α dependent repression of CPT1A in ccRCC interferes with fatty acid transport into the mitochondria and induces the accumulation of fatty acids and lipid droplets. In fact, tumor growth was suppressed upon reconstitution of CPT1A in VHL-deficient ccRCC in vivo [146].

Perilipin 2 (PLIN2), a lipid droplet coat protein, is upregulated in ccRCC via a HIF-2α dependent mechanism. Depletion of HIF-2α in 786-O and RCC4 cells using shRNA constructs reduces PLIN2 mRNA and protein expression whereas HIF-1α silencing increases PLIN2 mRNA and protein levels in RCC4 cells. Furthermore, HIF-2α knockdown reduces tumor growth in ccRCC xenografts which could be partially restored by exogenous PLIN2 expression. Moreover, PLIN2 mediated lipid storage reduces ER stress, thereby, maintaining ER integrity that otherwise would have been disrupted due to elevated protein synthesis in ccRCC. shRNA mediated knockdown of PLIN2 in 786-O and A498 cell lines renders the tumor cells vulnerable to oleic acid–induced cell death which is consistent with decreased ability to store lipids within LDs. Changes in ER morphology, activation of the UPR sensors PERK, IRE1α, and ATF6 and induction of multiple UPR target genes, characteristic of ER stress, results from PLIN2 and HIF-2α depletion and restored by exogenous PLIN2 expression [147]. Thus, HIF-2α mediated PLIN2 overexpression promotes tumor proliferation and survival (Figure 3). In addition, metabolic reprogramming in VHL-defective RCC cells involves the metabolism of glutamine to generate citrate and lipids through reductive carboxylation (RC) of α-ketoglutarate. In fact, transcriptionally active but pVHL insensitive HIF-2α expression in pVHL reconstituted 786-O cells could induce RC. Furthermore, inhibitors of glutaminase, an enzyme that breaks down glutamine, renders VHL-deficient RCC cells sensitive to glutamine depletion in vitro, blocking tumor xenograft growth in mice. These data demonstrate the ability of HIF-2α to activate RC and its role in modulating lipogenesis in ccRCC [148].

### 3.7. MET

The tyrosine kinase receptor MET is frequently dysregulated in ccRCC and linked to poor prognosis and reduced survival. The MET receptor and its ligand, hepatocyte growth factor (HGF) is overexpressed in ccRCC, suggesting a possible role in renal oncogenesis [149,150,151]. MET is activated by auto-phosphorylation when bound by HGF [152,153,154] engaging several downstream signaling networks, including the PI3K/AKT/mTOR, phospholipase C (PLC), and RAS/MAPK/RAF/ERK pathways involved in tumor growth and invasion [154,155,156,157] (Figure 3). VHL inactivation in ccRCC leads to the constitutive activation of MET without the requirement for HGF mediated autophosphorylation since re-expression of wild type VHL reduced MET activity [158].

MET overexpression in VHL-deficient ccRCC is associated with an epithelial to mesenchymal transition (EMT) by inducing the expression of beta-catenin, N-cadherin and vimentin (Figure 3) while suppressing the expression of E-cadherin, indicating a potential involvement in metastasis. Attenuation of MET function by the pharmacologic inhibitor K252a reduced RCC growth in vitro and in a nude mouse model [158,159]. Overexpression of MET may be the primal causative event in tumor invasion and metastasis induced by VHL loss suggesting its utility as a potential downstream candidate in the context of HIF-α overexpression [159,160].

The MET/HGF pathway can also regulate VEGF expression and promote angiogenesis during tumor progression [161]. Indeed, MET expression is upregulated in chronic sunitinib treatment and promotes resistance to sunitinib by inducing angiogenesis and prometastatic behavior [162] (Figure 3). Enhanced MET activity stimulates angiogenesis by activating ERK and PI3K/AKT signaling and inducing secretion of VEGF. Furthermore, MET induces EMT via upregulation of Snail and beta-catenin to support ccRCC invasion and metastasis. Consistent with these findings, RNA interference mediated or pharmacological inhibition of MET in sunitinib treated 786-O ccRCC reduced metastatic behavior and restored susceptibility to sunitinib [162].

Finally, overexpression of MET is associated with the upregulation of programmed death-ligand 1 (PD-L1) in pre-clinical models and sunitinib-treated metastatic RCC patients, implying its possible role in regulating immune checkpoint therapy in ccRCC [163,164] (Figure 3). Since PD-L1 inhibits antitumor T-cell immunity, the MET pathway may facilitate escape from immune-mediated cytotoxicity thereby facilitating the increased survival of RCC [163,164,165,166].

### 3.8. AXL

AXL, a tyrosine kinase receptor is upregulated in ccRCC. AXL is a direct target of HIF-2α and its overexpression correlates with ccRCC tumor invasion and metastasis (Figure 3). In fact, the invasive and metastatic phenotype in ccRCC was attenuated upon genetic and therapeutic inactivation of AXL in vivo [167]. AXL also forms a complex with the non-receptor tyrosine kinase SRC and mediates the enhanced activity of MET in an HGF-independent manner to facilitate tumor migration and invasion in ccRCC [167]. AXL activity in sunitinib insensitive ccRCC, moreover, supports its involvement in drug resistance. Overexpression of AXL and MET in sunitinib resistant ccRCC cells is associated with upregulation of Snail and beta-catenin (thereby regulating EMT or epithelial plasticity) indicative of increased cell migration and invasion. Indeed, inactivation of AXL via cabozantinib decrease metastatic behavior in vitro and restored drug susceptibility of RCC xenografts [162].

### 3.9. MT1-MMP

Membrane type-1 matrix metalloproteinase (MT1-MMP), a matrix-degrading enzyme, is transcriptionally induced upon HIF-2α stabilization in VHL-negative ccRCC cell lines [168] (Figure 3). MT1-MMP localizes to the leading edge of invasive cells to cleave extracellular matrix (ECM) molecules including fibronectin, lamnin-1 and 5, and fibrin as well as collagen type I, II and III, all of which are characteristics of tumor invasion and metastasis [169].

Upregulation of MT1-MMP is linked to the aggressive behavior of various cancers [170,171]. MT1-MMP overexpression in the VHL-deficient ccRCC cell line pRc-9 promotes the degradation of type I collagen and induces invasiveness. The wild type VHL reconstituted ccRCC cell line WT8, moreover, exhibits enhanced collagen degradation and dissemination upon upregulation of either HIF-2α or MT1-MMP while silencing of MT1-MMP by RNAi blocked the invasiveness of ccRCC cells [172].

### 3.10. PI3K/AKT/mTOR

The PI3K/AKT/mTOR network is a key contributor to major pathways that control cell growth, differentiation, migration, survival, angiogenesis, and metabolism and is commonly deregulated in several malignancies including ccRCC. Overactivity of mTOR signaling is frequently observed in VHL-deficient ccRCC and related to an aggressive tumor phenotype and poor survival in ccRCC patients [173,174]. AKT/mTOR mutations are evident in ccRCC, leading to its hyperactivation [175]. Moreover, mTORC1 activity is regulated via the amino acid carrier SLC7A5 (LAT1) in a HIF-2α dependent mechanism (Figure 3). mTORC1 activity and SLC7A5 expression are repressed upon HIF-2α silencing in 786-O VHL-deficient renal carcinoma cells growing under reduced amino acid availability (essential amino acids and glutamine). Interestingly, SLC7A5 silencing in 786-O cells to levels similar to those obtained after HIF-2α suppression attenuated mTORC1 activity and reduced xenograft size, consistent with the critical tumor-promoting properties of HIF-2α [176]. DEPTOR, an endogenous inhibitor of mTORC1 activity, is dramatically suppressed in VHL-deficient ccRCC cell lines as well as ccRCC patient samples, suggesting one mechanism for increasing mTORC1 activity during ccRCC progression. DEPTOR is potentially downregulated via both isoforms of HIF-α as genetic and chemical inhibition of HIF-2α resulted in a marked increase in DEPTOR mRNA and protein level in both the 786-O and RCC4 cell lines. Knockout of DEPTOR, moreover contributed to enhanced tumor cell proliferation in VHL competent cells, whereas, restoration of DEPTOR expression in ccRCC cells inhibited tumor growth [177].

### 3.11. Collective Hierarchical Actions of HIF-2α Target Genes

HIF-2α target genes collectively promote numerous pro-oncogenic changes (e.g., cell proliferation, epithelial to mesenchymal transition (EMT), invasion/metastasis, angiogenesis, metabolic reprogramming, immune tolerance, and extracellular matrix degradation) linked to kidney cancer progression. Hierarchical orders of oncogenic responses initiated by the deregulation of VHL-HIF-2α axis during ccRCC are depicted in Figure 3. Elevated HIF-2α, for example, leads to the induction of EGFR and TGF-α contributing to cell proliferation and tumor growth [78,79,80,82,83]. Overexpression of c-Myc and CCND1 downstream of HIF-2α also promote cell cycle progression and oncogenesis [54,88,126,128]. The major HIF-2α target gene, MET and its ligand hepatocyte growth factor (HGF) are both overexpressed in ccRCC and induces EMT by promoting the expression of beta-Catenin, N-Cadherin and vimentin [150,151,159,160]. Moreover, MET activation initiates several downstream signaling pathways, such as PI3K/AKT/mTOR, phospholipase C (PLC), and RAS/MAPK/RAF/ERK [157] providing a potential mechanism leading to cancer cell proliferation, angiogenesis, invasion, and metastasis. MET also facilitates increased survival of renal cancer cells through the upregulation of programmed death-ligand 1 (PD-L1) expression [163,164]. HIF-2α is an upstream regulator of VEGF induction, which promotes tumor angiogenesis and ccRCC progression [107,108,109]. MET/HGF pathway also regulates VEGF expression and promotes angiogenesis during invasion and metastasis [161], reflecting additional levels of genetic crosstalk and hierarchy during progressive ccRCC. AXL, a tyrosine kinase receptor, is a direct target of HIF-2α and its overexpression is correlated with ccRCC invasion and metastasis [167]. Tumor GLUT-1 induction is associated with a metabolic switch from aerobic glycolysis and mitochondrial oxidative phosphorylation to anaerobic glycolysis, which facilitates neoplastic growth [135,136,137]. Induction of Membrane type-1 matrix metalloproteinase (MT1-MMP) upon HIF-2α stabilization is possibly linked to tumor invasion via the degradation of type I collagen matrix likely supporting tumor invasion [171]. The clear cell morphology in ccRCC is due to the high lipid accumulation, induced by carnitine palmitoyltransferase 1A (CPT1A) repression and Perilipin 2 (PLIN2) overexpression via HIF-2α dependent mechanisms [145,146]. Moreover, PLIN2 mediated lipid storage promotes tumor proliferation and survival via maintaining the ER integrity during elevated protein synthesis in ccRCC [146]. Therefore, HIF-2α-mediated changes to glucose and lipid metabolism can sustain kidney tumor progression. MET and AXL overexpression or activation is linked to drug resistance to VEGF inhibitors [161], indicating that certain downstream targets of HIF-2α collectively function to retain oncogenic behavior.

## 4. Current Treatments, Medical and Scientific Challenges to ccRCC

Patients with localized RCC commonly undergo surgical procedures including partial or radical nephrectomy depending on the stage of the disease (such as favorable or intermediate-risk disease) and their overall health. Active surveillance and ablative therapies are favored for patients who are not candidates for surgical intervention [2,17,19,21,22,178]. Cytokine–based therapy such as interferon-α and high dose IL-2 (Interleukin-2) exhibit clinical activity against RCC and was the primary treatment of choice for patients with advanced and metastatic RCC. However, the use of cytokine based immune therapies are limited due to their significant toxicity and low response behavior [179]. Investigation into RCC molecular biology contributed to the development of targeted therapies targeting the VEGFR (VEGFR-tyrosine kinase inhibitors-VEGF-TKI- such as Sunitinib, Pazopanib, Sorafenib, Axitinib, Cabozantinib, Lenvatinib), VEGF (anti-VEGF antibodies such as Bevacizumab) and mTOR pathways (e.g., Everolimus and Temsirolimus) and were approved by the FDA in the treatment of RCC patients [112,113,180,181,182,183,184,185,186,187,188,189,190,191] (Table 1). Recently, FDA approved Belzutifan, a small molecule inhibitor of HIF-2α, for patients with VHL disease associated tumors such as renal cell carcinoma (patients harboring localized disease), central nervous system hemangioblastomas, or pancreatic neuroendocrine tumors (Table 1) [192].

According to American Society of Clinical Oncology (ASCO) recent guidelines, risk stratification-based first line treatment protocols are recommended for metastatic RCC patients who are eligible for systemic therapy (not cytoreductive surgery) [193]. Metastatic RCC patients who meet favorable-risk stratification (0) could be offered a combined VEGFR- tyrosine kinase inhibitor (VEGFR-TKI) and immune checkpoint inhibitor (ICI) treatment regimen, while patients with pre-existing medical issues who still meet favorable-risk criteria may be eligible for monotherapy (either VEGFR-TKI or ICI inhibitor). Patients stratified as intermediate-risk (1–2) or poor-risk (3+) could receive the combined regimen of two ICI inhibitors (e.g., Nivolumab + Ipilimumab) or a VEGFR-TKI in combination with an ICI as the first line therapy [193]. Although mTOR inhibitor monotherapy is no longer considered the frontline treatment option for patients with advanced or metastatic RCC requiring systemic treatment, a combination of VEGFR-TKI and mTOR Inhibitor (e.g., Lenvatinib + Everolimus) may be used in the management of patients with progressive disease, who received VEGFR-TKI and ICI combination as the first line therapy [194]. 

Immune checkpoint inhibitors (ICI), such as PD-1 (programmed cell death-1) and PD-L1 (programmed cell death ligand-1), in the context of RCC has revolutionized treatment strategies [195]. The interaction between PD-1 and its ligand PD-L1 allows tumor cells to evade immune attack by preventing T cell activity in the tumor microenvironment [196]. To date, several anti-PD-1 monoclonal antibodies (mAbs) including Nivolumab [197,198,199], Pembrolizumab [200,201,202] and anti-PD-L1 mAbs such as Avelumab [203,204] have shown promising clinical efficacy in treating RCC patients either alone or in combination with other agents (VEGFR-TKI or anti-CTLA-4 antibody) (Table 1). The immune checkpoint protein, Cytotoxic T lymphocyte-associated antigen-4 (CTLA-4) is highly expressed in activated CD8+ T cells and contributes to tumor immunosuppression via interfering with the binding of CD28 with B7, leading to T cell inactivation [205]. Targeting CTLA-4 has received extensive attention and is the first FDA-approved immunotherapy in the treatment of metastatic melanoma [206]. Although Ipilimumab, an anti-CTLA-4 mAb, enhances anti-tumor immunity in metastatic RCC, the resulting autoimmune consequences prevented its development as monotherapy [207]. However, CTLA-4 blockade in combination with Nivolumab (a PD-1 inhibitor) have the necessary efficacy and safety to be offered as an alternative for patients with advanced RCC [208,209,210] (Table 1). Dual treatment regimens including the combination of Nivolumab and Ipilimumab (a CTLA-4 inhibitor) as well as the combination of Axitinib, (a VEGF-TKI) with the PD-1 inhibitors (e.g., Pembrolizumab, or Avelumab) have improved efficacy in ccRCC patients compared to the single treatment regimen alone [211,212,213,214,215,216,217,218,219] (Table 1).

Such modalities include use of anti-angiogenic drugs, directed largely at the VEGF-A pathway in combination with immunotherapy to target immune checkpoint inhibitors [220]. The benefit of anti-angiogenic approaches to cancer treatment, however, has been largely modest since tumor vascularization is a complex process that varies as a function of tumor type and anatomic localization [221]. Indeed, mechanisms of blood vessel formation in the cancer microenvironment involve sprouting as well as intussusceptive angiogenesis, recruitment of endothelial progenitor cells, vasculogenic mimicry and transdifferentiation of cancer stem cells to endothelial-like elements [221]. Several such heterogenous processes may occur within a single neoplasm complicating appropriate targeting strategies. While an additional confounder involves the realization, from single cell transcriptome analyses, that there are significant differences in endothelial cells derived from tumor vs. normal tissue, these studies may lead to the discovery of new therapeutically-useful targets [222,223]. These are potentially clinically important efforts since many patients fail to respond, or acquire resistance, to anti-angiogenic drugs. Moreover, there are considerable nephrotoxicity and cardiovascular side effects associated with anti-VEGF/VEGFR therapies that may be avoided by increasing the repertoire of new targetable genes and pathways. Indeed, a recent report has identified a prostate-specific membrane antigen (PSMA) as a specific marker of tip-like endothelial cells in a large number of tumor types [222]. Tip-like endothelial cells are critical in the angiogenic response and constitute the major differential endothelial subcluster that distinguishes tumor from normal tissues. Such approaches may well lead to the development of more efficacious anti-angiogenic modalities with or without the combination of immune checkpoint targeting for the treatment of highly angiogenic tumor types (e.g., renal cell carcinoma). Overall, the management of RCC remains challenging due to the lack of validated biomarkers in deciding and planning patient treatment [2,224] and the development of drug resistance [225,226,227,228,229]. Furthermore, resurgence of RCC after surgery in a proportion of patients is common [23,230]. Small renal masses, moreover, do not produce any symptoms in many cases and remain undetected [21,231].

**Table 1:** List of FDA approved therapy to treat advanced renal cell carcinoma patients.

Target	Therapy	Citations
VEGFR	Sorafenib	[113]
	Sunitinib	[112,182]
	Pazopanib	[183]
	Cabozantinib	[184]
	Axitinib	[185,186]
VEGFR + mTOR	Lenvatinib + Everolimus (anti-mTOR)	[187]
VEGF	Bevacizumab + IFN-α (Cytokine)	[188,189]
mTOR	Temsirolimus	[190]
	Everolimus	[191]
PD-1	Nivolumab	[197,198,199]
	Pembrolizumab	[200,201,202]
PD-1 + CTLA-4	Nivolumab + Ipilimumab (anti-CTLA-4)	[210,211,232]
PD-1 + VEGFR	Nivolumab + Cabozantinib (anti-VEGFR)	[212,213,214,215]
	Pembrolizumab + Axitinib (anti-VEGFR)	[216,217,218]
	Pembrolizumab + Lenvatinib (anti-VEGFR)	[219]
PD-L1 + VEGFR	Avelumab + Axitinib (anti-VEGFR)	[203,204]
HIF-2α	Belzutifan	[192]

## 5. Conclusions

Hypoxia Inducible Factor-2α drives numerous pathways that favor ccRCC proliferation, progression, metastasis, and survival and its expression correlates with poor patient prognosis and survival. The HIF-α isoforms HIF-1α and HIF-2α exert opposing effects on ccRCC progression with HIF-1α acting as a tumor suppressor whereas HIF-2α exerts pro-oncogenic potential. HIF-α stabilization is an early event in the formation of renal lesions which overtime advance to malignant cancers because of multiple secondary events including the loss of the HIF-1α isoform. Although several agents are directed to HIF-2α and associated pathways, the development of drug resistance is a major contributor to the low survival rate of ccRCC patients. Further research focusing on the HIF-2α signaling network may well define new targets and therapeutic opportunities for disease management. It should be noted that HIF-2α-independent but VHL-dependent signaling networks also likely contribute to RCC progression. Such targets include tumor suppressor p53, transcription factor, NF-κB and retinoblastoma protein, Rb which can modulate apoptosis, cell survival and senescence phenotypes downstream of VHL deregulation [233]. Additional studies are necessary to establish functional interactions between HIF-2α-independent and -dependent networks in the progression of kidney cancer.
